# Enhancement of violaxanthin accumulation in *Nannochloropsis oceanica* by overexpressing a carotenoid isomerase gene from *Phaeodactylum tricornutum*

**DOI:** 10.3389/fmicb.2022.942883

**Published:** 2022-08-31

**Authors:** Yan Sun, Yi Xin, Luyao Zhang, Ying Wang, Ruolan Liu, Xiaohui Li, Chengxu Zhou, Lin Zhang, Jichang Han

**Affiliations:** ^1^College of Food and Pharmaceutical Sciences, Ningbo University, Ningbo, China; ^2^State Key Laboratory of Marine Resource Utilization in the South China Sea, College of Oceanology, Hainan University, Haikou, China; ^3^Key Laboratory of Applied Marine Biotechnology of Ministry of Education of China, School of Marine Science, Ningbo University, Ningbo, China; ^4^College of Marine Life Science, Ocean University of China, Qingdao, China

**Keywords:** violaxanthin, carotenoid isomerase, transgenic manipulation, *Nannochloropsis oceanica*, *Phaeodactylum tricornutum*

## Abstract

*Nannochloropsis* has been considered as a promising feedstock for the industrial production of violaxanthin. However, a rational breeding strategy for the enhancement of violaxanthin content in this microalga is still vacant, thereby limiting its industrial application. All-*trans*-lycopene locates in the first branch point of carotenogenesis. The carotenoid isomerase (CRTISO), catalyzing the lycopene formation, is thus regarded as a key enzyme for carotenogenesis. *Phaeodactylum tricornutum* can accumulate high-level carotenoids under optimal conditions. Therefore, it is feasible to improve violaxanthin level in *Nannochloropsis* by overexpression of *PtCRTISO*. Protein targeting analysis of seven PtCRTISO candidates (PtCRTISO1–6 and PtCRTISO-like) demonstrated that PtCRTISO4 was most likely the carotenoid isomerase of *P*. *tricornutum*. Moreover, the transcriptional pattern of *PtCRTISO4* at different cultivation periods was quite similar to other known carotenogenesis genes. Thus, *PtCRTISO4* was transformed into *N*. *oceanica*. Compared to the wild type (WT), all three transgenic lines (T1–T3) of *N*. *oceanica* exhibited higher levels of total carotenoid and violaxanthin. Notably, T3 exhibited the peak violaxanthin content of 4.48 mg g^–1^ dry cell weight (DCW), which was 1.68-folds higher than WT. Interestingly, qRT-polymerase chain reaction (PCR) results demonstrated that phytoene synthase (*NoPSY*) rather than ζ-carotene desaturase (*NoZDS*) and lycopene β-cyclase (*NoLCYB*) exhibited the highest upregulation, suggesting that *PtCRTISO4* played an additional regulatory role in terms of carotenoid accumulation. Moreover, *PtCRTISO4* overexpression increased C18:1n-9 but decreased C16:1n-7, implying that C18:1 may serve as a main feedstock for xanthophyll esterification in *Nannochloropsis*. Our results will provide valuable information for the violaxanthin production from *Nannochloropsis*.

## Introduction

Carotenoids are a family of terpenoid pigments with a common C40 methyl-branched hydrocarbon backbone (Takemura et al., 2021). Based on the absence or presence of oxygen atom, carotenoids are classified into carotenes (without oxygen) and xanthophylls (with oxygen). Up to now, around 1,100 types of natural carotenoids have been described, and many of them possess multiple benefits for human health (Ren et al., 2021). Violaxanthin is one type of valued xanthophylls showing a wide range of potential applications in food, pharmaceutical, and cosmetic sectors due to its anti-inflammatory, anti-photoaging, anti-oxidative, anti-lipid peroxidation, and anti-proliferative activities (Koller et al., 2014; Wang et al., 2018; Park et al., 2021). However, the industrial production of violaxanthin is still inaccessible due to the absence of appropriate organism resources presently (Park et al., 2021; Takemura et al., 2021).

*Nannochloropsis* is a genus of unicellular microalgae belonging to phylum Ochrophyta, class Eustigmatophyceae, and is well known for rich eicosapentaenoic acid (EPA, C20:5n-3) and lipid contents (Xin et al., 2017; Han et al., 2020). Because *Nannochloropsis* can tolerate broad environments, even wastewater or blowing flue gas (Dong et al., 2014; Zhu et al., 2014), it is of great interest to develop *Nannochloropsis* for commercial uses. Additionally, *Nannochloropsis* is also famous for its abundant violaxanthin, and has been taken as one of the most promising feedstocks for the scaled-up production of violaxanthin due to the advantages mentioned above (Park et al., 2021). Up to now, many studies aiming to improve the violaxanthin productivity of *Nannochloropsis* has been carried out by abiotic conditions modulation (Ma R. et al., 2018; Chua et al., 2020), trophic modes alteration (Menegol et al., 2019), and nutrients adjustment (Neto et al., 2018). However, the breeding of elite strains *via* transgenic approaches has rarely been tested in this field although the techniques for gene stacking, and targeted gene disruption and repression in the *Nannochloropsis* have been well developed (Wang et al., 2021).

**FIGURE 1:**
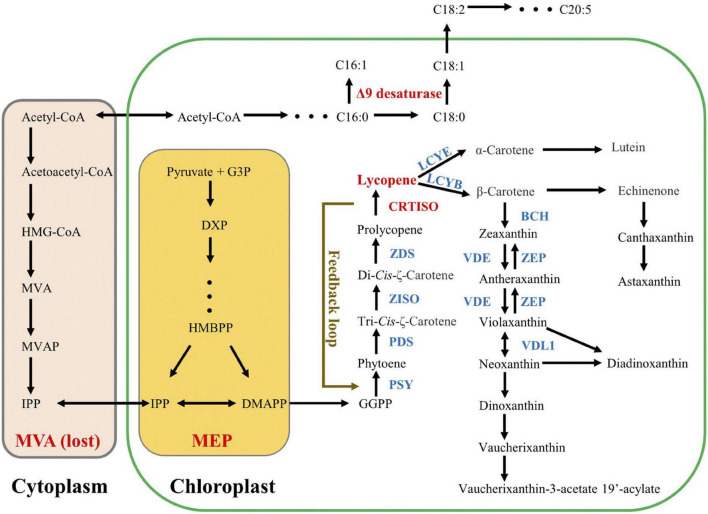
Hypothetic carotenoid pathway in *Nannochloropsis*. Lycopene is the first branch point in carotenoid biosynthesis pathway. Acetyl-CoA is the common precursor for both MVA pathway and fatty acid pathway, and *Nannochloropsis* had lost the former one. Both C16:1 and C18:1 are catalyzed by Δ9 desaturase. The presence of a feedback loop between CRTISO and PSY is proposed by our data.

The biosynthesis of carotenoids has been widely studied in both higher plants and microalgae *via* characterization of key genes in model organisms (Dautermann et al., 2020). Carotenes possessing the formula C_40_H_56_ are the precursors or intermediates of downstream xanthophylls (Huang et al., 2017). Similar to higher plants, the biosynthesis of carotenes in microalgae also start from the C5 substances of isopentenyl diphosphate (C_5_H_12_O_7_P_2_, IPP) and dimethylallyl diphosphate (C_5_H_12_O_7_P_2_, DMAPP) (Grassi et al., 2013; Dautermann et al., 2020; Figure 1). In the first step, three sequential condensation reactions, which consume one DMAPP molecule and three IPP molecules, generate geranylgeranyl diphosphate (C_20_H_45_N_3_O_7_P_2_, GGPP) (Morrone et al., 2010). Then PSY converts two GGPP molecules into the colorless phytoene (Bertrand, 2010). Subsequently, a multistep enzymatic process, carried out by phytoene desaturase (PDS), ζ-carotene isomerase (Z-ISO), ZDS, and CRTISO, introduces four conjugated double bonds into phytoene, generating all-*trans*-lycopene. After that, the pathway splits into the synthesis of either α-carotene or β-carotene, catalyzed by lycopene ε-cyclase (LCYE) or LCYB, respectively. Finally, with β-carotene as the substrate, various xanthophylls are successively synthesized. Since lycopene functions as an important branch point in the carotenoid biosynthesis pathway, the role of CRTISO (catalyzing the final step of lycopene formation) on carotenoid metabolism has been widely elucidated in higher plants (Kato et al., 2007; Vallabhaneni and Wurtzel, 2009; Galpaz et al., 2013; Su L. et al., 2015). For example, the *CRTISO*-knockout in *Brassica napus* resulted in decrease of xanthophylls but increase of phytoene and phytofluene (upstream carotene of lycopene), suggesting the crucial role of CRTISO in xanthophylls accumulation (Li et al., 2022). Meanwhile, the mutation of CRTISO in Chinese cabbage accompanied by the color change of the inner head leaves from white to orange (Su T. B. et al., 2015). Such a color change caused by CRTISO functional loss has also been obtained from other organisms, e.g., rice, tomato, melon, and watermelon (Isaacson et al., 2002; Chai et al., 2011; Fantini et al., 2013; Galpaz et al., 2013; Grassi et al., 2013). Moreover, the mutation/overexpression of *CRTISO* was reported to induce significant transcript alteration of other carotenoid genes (both upstream and downstream genes), suggesting a potential regulatory role in carotenoid biosynthesis pathway (Vanessa et al., 2013; Su T. B. et al., 2015; Sun et al., 2020). In addition, CRTISO has impacts on the amounts of Photosystem II (PSII) core proteins as well (CP43, CP47, and D1) (Fang et al., 2010; Wei et al., 2010). Compared to comprehensive understanding in higher plants, microalgal CRTISO is rarely reported. In *Picochlorum celery*, *CRTISO*-knockout resulted in more than 50% reduction of lutein content compared to the WT (Krishnan et al., 2020), suggesting that CRTISO played a key role in carotenoids accumulation of microalgae. Nevertheless, it is unknown to rationally increase carotenoid level by regulation of *CRTISO* expression in microalgae.

As a model diatom featured with rich carotenoids, *Phaeodactylum tricornutum* genome was predicted to harbor seven CRTISO candidates (Coesel et al., 2008; Kadono et al., 2015; Gaidarenko et al., 2020). In the present study, the subcellular localizations of seven PtCRTISO candidates were analyzed by various bioinformatics tools first, and the results demonstrated that PtCRTISO4 was the most likely protein responsible for the transformation of all-*trans*-lycopene from prolycopene. Thus, *PtCRTISO4* was cloned and heterologously expressed in *N. oceanica* IMET1. According to the phenotypic and transcriptional discrepancy between the WT and transformants, the role of *PtCRTISO4* in microalgae growth and carotenoid accumulation was evaluated.

## Materials and methods

### Microalgae strains and cultivation conditions

Cells of both *N. oceanica* IMET1 and *P. tricornutum* Bohlin CCMP2561 under early stationary growth phase were inoculated into fresh f/2 liquid medium (Guillard and Ryther, 1962) with an inoculation ratio of 1:9, and cultivated at 25^°^C under a photoperiod of 12 h light/12 h dark with a light intensity of 60 μmol photons m^–2^ s^–1^.

### Identification of potential key *PtCRTISO*

Endoplasmic reticulum (ER) signal peptides of seven PtCRTISO candidates were analyzed using TargetP-2.0 (Emanuelsson et al., 2000), Signal-3L 2.0 (Shen and Chou, 2007; Zhang and Shen, 2017), SignalP-5.0 (Armenteros et al., 2019), HECTAR (Gschloessl et al., 2008), and IPSORT (Hideo et al., 2002) first. Subsequently, the signal peptides were manually cut off, and the shortened sequences were analyzed again using IPSORT to further identify the subcellular localization. All sequences of *P. tricornutum* were obtained from the database of Ensembl Protists.

*P. tricornutum* at Day 6, 9, 12, and 15 were collected by centrifugation (5,000 g, 10 min), and the pellets were used for total RNA extraction using Plant RNA kit (Omega, United States) according to the manufacturer protocol. The quality and quantity were estimated by agarose gel electrophoresis primarily, and further determined based on the A230, A260, and A280 measured by a NanoDrop ND1000 (NanoDrop, United States). After gDNA removal and cDNA synthesis using PrimeScript™ RT Reagent Kit (Takara, China), qRT-polymerase chain reaction (PCR) (Bio-Rad, United States) was carried out to investigate the transcript patterns of four genes: *PtCRTISO4*, *PtPSY*, *PtZEP2* (encoding zeaxanthin epoxidase 2), and *PtVDL1* (encoding violaxanthin de-epoxidase-like 1) during the whole cultivation period. *CdkA* (encoding cyclin dependent kinase) was used for the normalization of genes expression using the 2^–Δ^ ^Δ^ *^CT^* method. Primer pairs were designed using Primer Premier 5 and listed in Supplementary Table 1. In ahead of conducting qRT-PCR, conventional RT-PCR under the same reaction conditions was adopted to verify the specificity of primers.

### Gene isolation and vector construction

*P. tricornutum* under mid-exponential phase was collected for total RNA isolation. Specific primers of F-*Nde*I-*PtCRTISO4* (containing *Nde*I site) and R-*Pst*I-*PtCRTISO4* (containing *Pst*I site) were used to acquire the full length *PtCRTISO4* (Table 1) based on gDNA-free cDNA. The PCR was performed for 30 thermal cycles with a program of 95^°^C for 30 s, 95^°^C for 5 s, 55^°^C for 30 s, and 65^°^C for 5 s, and included a final extension step for 10 min at 72°C. After being digested with *Nde*I and *Pst*I restriction enzymes, the amplified *PtCRTISO4* fragment was ligated into pXJ450 generating the overexpression vector designated as pXJ450-PtCRTISO4 (Zhang et al., 2022).

**TABLE 1:** Primers for *PtCRTISO4* isolation and positive transformants selection.

Primers	Primer sequence (5′–3′)	Product size
F-*Nde*I-*PtCRTISO4*	GGAATTCCATATGATGAGATTT TCGGAAAGATC	1,820 bp
R-*Pst*I-*PtCRTISO4*	TCCCCTGCAGAGACTTTTGTTC CTTTTCCGGTTGTTTTTCGGAG	
F-V1-*PtCRTISO4*	GCACACATCAGTCAGCAC	824 bp
R-V1-*PtCRTISO4*	GCAAAGCATATCTAGCCA	
F-V2-*PtCRTISO4*	ATGAGATTTTCGGAAAGATCAC	1,794 bp
R-V2-*PtCRTISO4*	AGACTTTTGTTCCTTTTCCGG	

The underline indicates the restriction enzyme cutting sites.

### Transformation of *Nannochloropsis oceanica* by electroporation

*Nannochloropsis* cells under mid-exponential growth stage were centrifugated at 5,000 g for 5 min. After being washed for three times using precooled 375 mM sorbitol solution (4°C), cells were re-suspended in 375 mM sorbitol solution and concentrated to 10^8^ cells L^–1^. Then, 200 μL cells suspension and 2 μg linearized pXJ450-PtCRTISO4 were gently mixed and incubated on ice for 20 min. The nuclear transformation was performed using a BTX ECM 630 electroporator (high-voltage of 11,000 V cm^–1^) (Xin et al., 2017). After the pulse, cells were immediately transferred to glass tube containing fresh medium and incubated under low light conditions for 48 h shaking at 120 rpm. Then, cells were plated on the solid f/2 medium (1% agar) containing 2.5 μg mL^–1^ zeocin. Around 3 weeks later, visible algal colonies were randomly picked and inoculated into f/2 medium for further verification.

### Polymerase chain reaction screening for the *PtCRTISO4* transformant

Genomic DNAs were isolated using HiPure SF Plant DNA Mini kit (Magen, China). Standard PCR was performed using specific primers of F-V1-*PtCRTISO4* and R-V1-*PtCRTISO4* (Table 1) to amplify the recombinant *PtCRTISO4*. WT and plasmid of pXJ450-PtCRTISO4 were used as the negative and positive control, respectively. After purification, the PCR fragments were sequenced. To further test whether the *PtCRTISO4* was transcribed, total RNA was extracted, quantified, and reverse-transcribed. Based on the gDNA-free cDNA, another pair of specific primers (F-V2-*PtCRTISO4* and R-V2-*PtCRTISO4*) were adopted to carry out standard PCR. After sequencing, positive transformants (T1–T3) were chosen for further analysis.

### Transcriptional and expressional verification of *PtCRTISO4* in *Nannochloropsis oceanica*

Cells of *N. oceanica* at Day 6 were collected by centrifugation (5,000 g, 10 min) for total RNA isolation using Plant RNA kit (Omega, United States) according to the manufacturer protocol. Then, primers of F-V2-*PtCRTISO4* and R-V2-*PtCRTISO4* were adopted to verify whether the *PtCRTISO4* was successfully transcribed in *N. oceanica* or not.

To verify the expression level of *PtCRTISO4*, proteins of all transformants were extracted using Plant Protein Extraction Kit (Solarbio, China) and concentrated to the same amount using Easy II Protein Quantitative Kit (Transgen, China). Then, the proteins were separated by SDS-PAGE and transferred to polyvinylidene fluoride membranes. After being washed using TBST buffer for three times, the membranes were blocked for 45 min in blocking buffer (Beyotime, China) and then incubated with mouse anti His-tag mAb (ABclonal, United States). Membranes were then washed six times using TBST buffer and then incubated with HRP Goat Anti-Mouse IgG (ABclonal, United States). Finally, WesternBright ECL HRP substrate (APGBio, China) and the ChemiScope 6100 (Clinx Sci, China) were used to detect the immunoreactive proteins. *PtGAPDH* (GenBank accession: XP_002184760) was served as the internal control, and GAPDH Rabbit Monoclonal Antibody (Beyotime, China) and HRP-labeled Goat Anti-Rabbit IgG (Beyotime, China) were used as the primary and secondary antibodies, respectively.

### Phenotyping determination of *Nannochloropsis oceanica*

Cells under early stationary growth phase were collected and concentrated to the same density (OD_750_ = 0.15), and then inoculated into f/2 medium with a ratio of 1:9 to conduct a batch cultivation. During the whole cultivation period, OD_750_ was monitored every 2 days by Thermo Fisher Scientific Microplater Reader (Varioskan LUX, Finland). The specific growth rate (μ) was calculated with the equation μ = (lnN_*t*_ − lnN_0_)/t_1_ − t_0_, in which N_*t*_ and N_0_ were OD_750_ values at time t_1_ and t_0_, respectively. The biomass was determined by GF/F filters using 10 mL microalgae cultures at the final day. PSII performance, including the maximal PSII quantum yield (*F*_*v*_/*F*_*m*_), non-photochemical quenching (NPQ), and photosynthetic electron transport rate (ETR) were determined by WATER-PAM (WALZ, Germany).

For carotenoids extraction, 5 mL chloroform/methanol (1/1, v/v) containing 0.1% butylated hydroxy toluene was mixed with 5 mg lyophilized algal powder (Day 16). After vortexing for 5 min, the carotenoids were extracted *via* ultrasonication for 20 min in the ice-water bath. Then, the mixture was centrifuged with 12,000 rpm at 4°C for 5 min, and the supernatant was filtered with a polytetrafluoroethylene membrane (0.22 μm). All operations were performed in the dark to avoid degradation of carotenoids. An ultra-high-pressure liquid chromatography coupled with a quadrupole-orbitrap high resolution mass spectrometer (Thermo Fisher, Quadrupole-Exactive) was used for carotenoid analysis. Liquid samples of 5.0 μL were injected into Syncronis C18 column (2.1 mm × 150 mm, 1.7 μm), and flow rate was set as 0.3 mL min^–1^. Acetonitrile: water (9:1) containing 10 mM NH_4_HCO_2_ was used as solution A, and 30% isopropanol in acetonitrile as solution B. The mass spectrometry analysis was same with the method reported previously (Li et al., 2021). The determination of carotenoids was accomplished by the software Exactfinder™ (Thermo Scientific) based on the ion *m*/*z* values (mass error ≤ 5 × 10^–6^), retention time, isotopic distribution, and MS/M spectra. A mixture including nine types of carotenoids standards (Sigma, United States), including β-carotene, zeaxanthin, antheraxanthin, violaxanthin, vaucheriaxanthin, diadinoxanthin, lutein, canthaxanthin, and astaxanthin, was used for the qualification and quantification analysis.

FA was extracted from 10 mg lyophilized algal powder (Day 16) using KOH-CH_3_OH (2 M, 3 mL) and HCl-CH_3_OH (3 M, 1.5 mL) successively. Details have been described in our previous report (Zhang et al., 2021). The FA methyl esters dissolved in *n*-hexane were further analyzed by a gas chromatography-mass spectrometry (GC-MS) (8890A-5977B, Agilent Technologies, United States) equipped with an auto-sampler (7963A), using a CD-2560 capillary column (100 m × 0.25 mm × 0.2 μm) (CNW, Germany). Highly pure helium supplied at a flow rate of 0.81 mL min^–1^ was used as the carrier gas. The oven temperature program began with 140°C for 5 min, and rose to 240°C for 20 min with a rate of 4^°^C min^–1^. The mass spectrometer was operated with electron compact mode at an ionization energy of 70 eV, and scanned from 50 to 600 *m*/*z*. The identification and quantification of FAs were accomplished according to the retention time, NIST14.L and Wiley7 databases.

### Transcript analysis of carotenoid biosynthesis genes of *Nannochloropsis oceanica*

Total RNA of cells at Day 16 was isolated. Then, the transcript abundances of nine genes: *NoPDS*, *NoPSY*, *NoLCYB*, *NoZDS*, *NoZEP2*, *NoVDE* (encoding violaxanthin de-epoxidase), *NoVDL*, *NoCP43*, and *NoCP47*, were investigated to evaluate the effect of *PtCRTISO4* on the regulation of carotenoids biosynthesis in *N. oceanica*. *Actin 1* (*ACT1*), *Actin 2* (*ACT2*), and *Tubulin alpha* (*TUA*) were employed as the candidate reference genes. Related sequences were obtained from the NanDeSyn (Gong et al., 2020), and primers were listed in Supplementary Table 1.

### Statistical analysis

All strains with three biological replicates were used in this study. Statistical analysis was performed by one-way analysis of variance (ANOVA) using SPSS Statistics 25.0, and the data were displayed as mean ± SD (standard deviation) (*n* = 3). The difference was considered statistically significant when *P* < 0.05 and extremely significant when *p* < 0.01.

## Results

### Analysis of *PtCRTISOs* candidates

Five types of software were employed to perform the N-terminal targeting signal analysis, and PtCRTISO4 was the only protein that the presence of signal peptide was supported by all bioinformatics tools. After the signal peptide being shortened manually, the subcellular localization of PtCRTISO4 was further analyzed using IPSORT, and the results demonstrated that PtCRTISO4 was targeted in chloroplast (Table 2). Besides that, we also found that PtCRTISO4 contained the GXGXXG motif, which is essential for the catalytic activity of carotenoid isomerase (CRTISO).

**TABLE 2:** Sequence analysis of PtCRTISOs candidates.

Protein	Protein ID	Signal peptide					Target	Motif
		Tar	Sig^1^	Sig^2^	IPS^1^	HEC	IPS^2^	GXGXXG
CRTISO1	Phatr3_J51868	+	+			+	–	+
CRTISO2	Phatr3_J54826						–	+
CRTISO3	Phatr3_J54842	+					–	
CRTISO4	Phatr3_J45243	+	+	+	+	+	Chlo	+
CRTISO5	Phatr3_J9210		+		+	+	Chlo	+
CRTISO6	Phatr3_J42980						–	
CRTISO-L	Phatr3_EG01981				+		–	+

Tar, TargetP-2.0; Sig^1^, SignalP-5.0; Sig^2^, Signal-3L; IPS^1^ and IPS^2^, analyzed by IPSORT with full length and shortened peptides, respectively; HEC, HECTAR. “+” means positive prediction result. “–” represents the targeted localization is mitochondria or cytoplasm. “Chlo” denotes the targeted localization is chloroplast.

**FIGURE 2:**
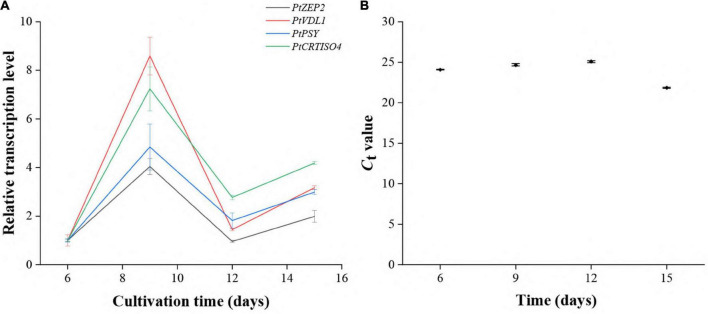
Expression patterns of five genes under different cultivation stages. **(A)** The transcriptional pattern of *PtCRTISO4* was quite similar to other three genes. **(B)** *C*_*t*_-values of *CdkA*.

The transcript abundances of *PtCRTISO4* as well as *PtPSY*, *PtZEP2*, and *PtVDL1* at different cultivation periods were investigated using qRT-PCR. The transcription levels of *PtCRTISO4* peaked at the ninth day, then fell at the twelfth day, and rose again at the fifteenth day (Figures 2A,B). Such a variation trend was quite similar to that of *PtPSY*, *PtZEP2*, and *PtVDL1*, suggesting that PtCRTISO4 was likely co-regulated with other three genes.

### Screening of positive transformants

**FIGURE 3:**
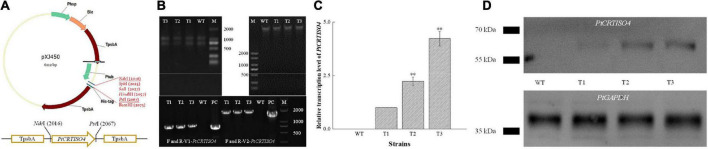
Construction of recombinant vector and screening of positive transformant. **(A)** After being digested by *Nde*I and *Pst*I, *PtCRTISO4* was inserted into pXJ450. **(B)** Genomic DNA and total RNA from transformants and of *N*. *oceanica*, and the polymerase chain reaction (PCR) results using primers of V1-*PtCRTISO4* and V2-*PtCRTISO4* based on gDNA and cDNA, respectively. M and PC indicate marker and positive control (recombinant plasmid), respectively. **(C)** Relative transcription levels of *PtCRTISO4.* **(D)** Western Blotting analysis using anti-His-tag antibody, *PtGAPDH* was used as the internal control. **Indicates a highly significant difference (*p* < 0.01).

The recombinant vector designated as pXJ450-PtCRTISO4 containing *TpsbA* promoter, *TpsbA* terminator, and *sh ble* (Figure 3A) was constructed and transformed into *N. oceanica*. After being screened by solid f/2 medium containing 2.5 μg mL^–1^ Zeocin, two pairs of specific primers: F/R-V1-*PtCRTISO4* (corresponding to part of the pXJ450 backbone and 5′ sequences of *PtCRTISO4* with an expected length of 824 bp) and F/R-V2-*PtCRTISO4* (flanking the full length of *PtCRTISO4* with an expected length of 1,794 bp) (Table 1) were used to verify the successful transformation based on total genomic DNA and gDNA-free cDNA, respectively. As shown in Figure 3B, the expected bands were obtained in all three transformants as well as in the plasmid of pXJ450-PtCRTISO4 but not in WT, demonstrating that the *PtCRTISO4* had been integrated into the genome of *N*. *oceanica*. Moreover, the qRT-PCR and western blotting results demonstrated that the *PtCRTISO4* in all three transgenic lines were successfully transcribed and translated, and T3 showed the highest transcriptional and expressional abundances (Figures 3C,D).

### Effect of overexpressed *PtCRTISO4* on the growth performance of *Nannochloropsis oceanica*

**FIGURE 4:**
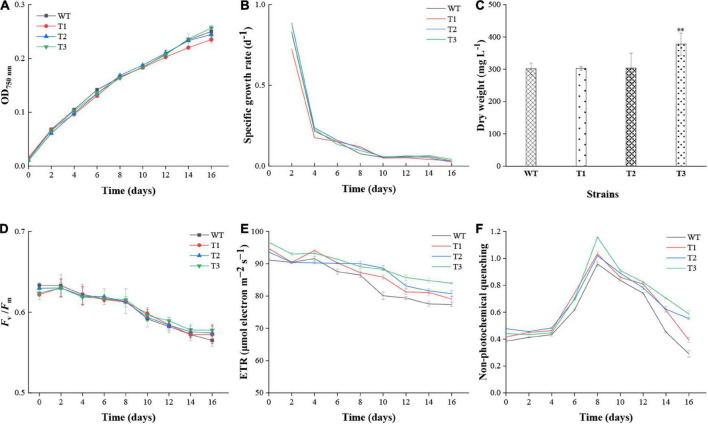
Growth performance and chlorophyll fluorescence parameters of WT and transformants. **(A–C)** Cell density, specific growth rate, and biomass of WT and transformants. **(D–F)** *F*_*v*_/*F*_*m*_, photosynthetic electron transport rate (ETR), and non-photochemical quenching (NPQ) of WT and transformants. **Indicates a highly significant difference (*p* < 0.01).

The effect of *PtCRTISO4* overexpression on the growth performance of *N*. *oceanica* was assessed based on OD_750_ and biomass. After 16 days cultivation, all *N*. *oceanica* strains reached their maximal cell concentrations. In details, T3 exhibited the highest OD_750_ of 0.26 ± 0.02, followed by WT with a value of 0.25 ± 0.01, both of which were slightly higher than T2 (0.24 ± 0.01) and T1 (0.23 ± 0.01) (Figure 4A). No significant differences were obtained between WT and transformants. The specific growth rates of all strains peaked at the second day, varying within the range of 0.72–0.89 d^–1^, and then drastically declined to around 0.21 d^–1^ at Day 4. After suffering a slow decrease from days 4 to 8, the values maintained at a relatively flat level around 0.05 d^–1^ until the end (Figure 4B). Except the first 2 days, the specific growth rates between WT and transformants showed no significant differences. The biomass of T3 at the final day was 377.78 ± 34.56 mg L^–1^, while other three strains with the values between 301.11 and 303.33 were quite close to each other, and significantly lower than the T3 (Figure 4C).

Various photophysiological parameters characterizing photosynthetic activity were measured to evaluate the effect of *PtCRITOS4* on the PSII of *N*. *oceanica*. Our data demonstrated that the *F*_*v*_/*F*_*m*_ ratios of all strains were similar to each other at all timepoints (Figure 4D). Besides that, all *F*_*v*_*/F*_*m*_ values displayed a common downward trend along with cultivation period, and reached below 0.6 since the tenth day, suggesting a severe nutrient deficiency appeared since that time. As for ETR, the maximum values nearly at all timepoints were obtained from T3, all of which were significantly higher than that of the WT (Figure 4E). The NPQ values of all transformants were higher than WT, demonstrating that the contents of xanthophyll cycle pigments (i.e., violaxanthin, antheraxanthin, and zeaxanthin) might be enhanced by *PtCRTISO4* (Figure 4F).

### Carotenoid analysis of wild type and transformants

**FIGURE 5:**
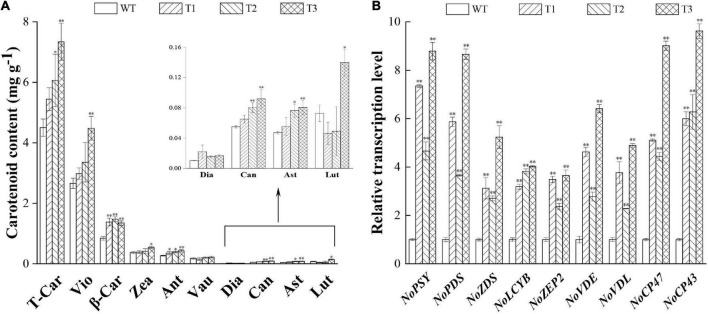
Comparison of carotenoid content and relative mRNA abundance between WT and transformants. **(A)** Carotenoid content of WT and transformants. **(B)** Transcription levels of the key genes. T-Car, total carotenoid content; Vio, violaxanthin; β-Car, β-carotene; Zea, zeaxanthin; Ant, antheraxanthin; Vau, vaucheriaxanthin; Dia, diadinoxanthin; Can, canthaxanthin; Ast, astaxanthin; Lut, lutein. * and ** indicate a significant (*p* < 0.05) and highly significant (*p* < 0.01) differences, respectively.

The overexpression of *PtCRTISO4* sharply enhanced the carotenoid accumulation of *N*. *oceanica*. All transformants displayed higher total carotenoid contents (varying within the range from 5.44 to 7.35 mg g^–1^ DCW) than the WT (4.49 mg g^–1^ DCW), and significant differences could be obtained from both T2 and T3 (Figure 5A).

In total, five pigments achieved a content over 0.1 mg g^–1^ DCW, among them β-carotene displayed the maximum increase ratio (64.29–75.00% higher than the WT). Such an observation can be partially attributed to the fact that β-carotene is the first major carotenoid downstream of lycopene (Gupta et al., 2021). Significant enhancement also could be obtained from the violaxanthin, though the increase magnitude was relatively smaller than that of the β-carotene. For example, the *N*. *oceanica* IMET1 reached a violaxanthin content of 2.66 mg g^–1^ DCW at Day 16, accounting for 59.24% of the total carotenoids (Figure 5A), while the transgenic lines were enhanced to 2.98–4.48 mg g^–1^ DCW. The highest violaxanthin was obtained from T3, of which the violaxanthin was 1.68-folds greater than the WT. Only small amounts of vaucheriaxanthin (0.15–0.22 mg g^–1^) were obtained in this study, and no significant alteration was observed between the WT and overexpressed lines (Figure 5A). Our data also indicated that the contents of both zeaxanthin and antheraxanthin (around one-tenth to violaxanthin) were also improved by the overexpression of *PtCRTISO4* (Figure 5A), what further resulted in the substantially increase of xanthophyll cycle activity featured by the enhancement of NPQ ratios (Figure 4F; Eilers et al., 2016; Chukhutsina et al., 2017). Besides that, four types of trace carotenoids, i.e., diadinoxanthin, canthaxanthin, astaxanthin, and lutein, were also detected from *N*. *oceanica*. As for the former three types, the transformants exhibited certain improvements, however, T1 and T2 were lower than the WT in terms of lutein.

### Transcript analysis of carotenegenesis genes in *Nannochloropsis oceanica*

To further reveal the mechanism of *PtCRTISO4* on regulating carotenoid accumulation, the expression levels of nine genes in *N*. *oceanica*, including seven carotenegenesis genes and two chlorophyll binding protein encoding genes, were compared between the WT and transformants. In ahead of conducting the comparison analysis, the stability of three candidate internal reference genes was determined first. Briefly, the expression levels of all three candidates (*C*_*t*_ values within the range of 21.26–26.27) were relatively steady in different strains. Among them, *TUA* exhibited the lowest coefficient of variation, and thus was chosen as the internal reference gene in this study (Supplementary Table 2).

The qRT-PCR analysis demonstrated that the transcriptions of seven genes relevant to carotenoid accumulation were significantly enhanced by the transformation of *PtCRTISO4*, and the maximum magnitude was yielded from T3 (Figure 5B). For example, the amounts of *NoPSY* and *NoVDL* in T3 were approximately 8.8 and 4.9 times higher than the levels obtained in WT, respectively. Such a huge enhancement of genes expression level in T3 coincided with its significant increase of carotenoid contents (Figure 5B). Moreover, the expression levels of CP43 and CP47 were also strengthened by the overexpression of *PtCRTISO4*.

### Fatty acid analysis of wild type and transformants

*Nannochloropsis* has been considered as a promising candidate for polyunsaturated fatty acids (PUFAs) production due to its high EPA contents (Gheysen et al., 2019; Wang and Jia, 2020; Zhang et al., 2021). Therefore, the FA contents and profiles were also analyzed. Our data revealed that the overexpression of *PtCRTISO4* did not induce significant change in total fatty acid (TFA) content. For example, the TFA content of WT was around 66.36 ± 3.89 mg g^–1^ DCW, and that of the transformants varied in the range from 60.92 ± 3.69 (T3) to 64.87 ± 0.37 (T2) mg g^–1^ DCW (Table 3). Our results also found that EPA was one of the predominant FAs in *Nannochloropsis* as well as the palmitic acid (C16:0) and palmitoleic acid (C16:1n-7), each of which accounted for 1/5–1/4 of the TFA. The contents of other two valuable PUFAs (i.e., linoleic acid, C18:2n-6; arachidonic acid, C20:4n-6) were similar with each other, varying from 4.93 ± 0.14% TFAs (C18:2 of T2) to 7.58 ± 0.03% TFAs (C20:4 of T2). These results were coincident with previous studies, indicating that *N. oceanica* was a promising source for EPA production (Gheysen et al., 2019; Wang and Jia, 2020; Zhang et al., 2021).

**TABLE 3:** Fatty acids analysis of WT and transformants.

Fatty acid	WT	T1	T2	T3
Myristic acid (C14:0)	5.11 ± 0.00	5.63 ± 0.71	6.54 ± 0.20	4.59 ± 0.34
Palmitic acid (C16:0)	20.53 ± 0.99	23.14 ± 0.90	21.85 ± 0.83	22.03 ± 0.66
Palmitoleic acid (C16:1n-7)	26.46 ± 2.67^a^	23.84 ± 0.71^b^	22.98 ± 0.16^b^	23.06 ± 0.30^b^
Hexadecanoic acid (C17:0)	0.43 ± 0.04	0.49 ± 0.03	0.53 ± 0.04	0.50 ± 0.00
Stearic acid (C18:0)	5.04 ± 0.65	2.94 ± 1.59	2.62 ± 2.06	4.52 ± 0.52
Oleic acid (C18:1n-9)	8.06 ± 0.39^b^	9.17 ± 0.25^a^	9.50 ± 0.21^a^	9.47 ± 0.29^a^
Linoleic acid (C18:2n-6)	6.07 ± 1.29	5.42 ± 0.74	4.93 ± 0.14	5.12 ± 0.12
Arachidonic acid (C20:4n-6)	6.68 ± 0.69	6.79 ± 0.25	7.58 ± 0.03	7.23 ± 0.34
Eicosapentaenoic acid (C20:5n-3)	21.61 ± 2.31	22.58 ± 0.70	23.47 ± 0.30	23.48 ± 0.14
SFA	28.70 ± 1.40	32.20 ± 1.23	30.02 ± 0.32	31.53 ± 0.79
MUFA	35.73 ± 2.28	33.01 ± 0.93	33.20 ± 0.24	32.58 ± 0.23
PUFA	34.26 ± 1.18	34.78 ± 0.33	36.78 ± 0.38	35.89 ± 0.57
SFA (mg g^–1^ DCW)	20.64 ± 1.32	21.36 ± 2.20	20.46 ± 0.84	19.28 ± 1.54
MUFA (mg g^–1^ DCW)	22.91 ± 0.22	21.90 ± 2.87	21.07 ± 0.66	19.82 ± 1.15
PUFA (mg g^–1^ DCW)	22.80 ± 3.22	23.08 ± 2.88	23.34 ± 1.48	21.83 ± 1.07
TFAs (mg g^–1^ DCW)	66.36 ± 3.89	66.33 ± 7.75	64.87 ± 0.37	60.92 ± 3.69

^a,b^Indicate statistical significance (p < 0.05).

As for FA profile, nine types of FAs, including four saturated fatty acids (SFAs), two mono unsaturated fatty acids (MUFAs), and three PUFAs, were obtained from both WT and transgenic strains (Table 3). All nine FAs showed no significant differences between WT and transformants except palmitoleic acid (C16:1n-7) and oleic acid (C18:1n-9). The C16:1 of WT (26.46 ± 2.67% of TFA) was 10.99–15.14% higher than three transformants (22.98 ± 0.30–23.84 ± 0.71% of TFA), while the C18:1 displayed an opposite variation trend.

## Discussion

### *PtCRTISO4* is the most likely carotenoid isomerase

Although the whole genome of *P*. *tricornutum* has been sequenced, the authentic CRTISO is still uncertain. Genomes of higher plants (e.g., tomato, carrot, and rice) usually harbor only one *CRTISO*, while corn genome harbors two *CRTISO* copies (Vanessa et al., 2013; Zhai et al., 2016). Surprisingly, seven putative *CRTISOs* have been proposed in *P*. *tricornutum* genome (Coesel et al., 2008; Kadono et al., 2015; Gaidarenko et al., 2020), implying that majority of these candidates may play other roles.

In diatoms, carotenegenesis proteins must across four membranes, the outermost of which is continuous with the ER (Gruber et al., 2007). The prediction results of all bioinformatics tools used in this study indicated that PtCRTISO4 possessed the ER signal peptides. Taking the ER signal peptides, chloroplast localization, and GXGXXG motif into consideration, we speculate that PtCRTISO4 is a CRTISO of *P*. *tricornutum*.

To consolidate this assumption, we further investigated the transcript levels of *PtCRTISO4* at different cultivation periods. It has been widely stated that many carotenogenesis genes are co-regulated (Sun et al., 2010; Ma X. et al., 2018; Yang et al., 2021). In this study, the transcriptional patterns of three functionally addressed genes (i.e., *PtPSY*, *PtZEP2*, and *PtVDL1*) and *PtCRTISO4* displayed the same variation trend during the whole cultivation process, suggesting that *PtCRTISO4* is likely co-regulated with other three genes and probably participates in carotenegenesis (Kadono et al., 2015; Kaur and Spillane, 2015). On basis of all results above, we decided to transform *PtCRTISO4* into *N*. *oceanica*.

### The overexpression of *PtCRTISO4* enhances the non-photochemical quenching and photosynthetic electron transport rate of *Nannochloropsis oceanica*

The final cell density, specific growth rates and *F*_*v*_*/F*_*m*_ of WT were similar to that of transformants, indicating a mild effect of *PtCRTISO4* on these parameters. However, the biomass accumulation can be significantly strengthened by *PtCRTISO4*. For example, the final biomass of T3 is around 1.25-folds higher than the WT. Such an observation can be attributed to the enhancement of ETR caused by the *PtCRTISO4* transformation. ETR represents the actual light utilization efficiency, and the higher ETR values mean the more electrons supplied for ATP generation and the higher biomass production (Lu et al., 2022). A previous study demonstrated that ETR is closely related to carotenoid accumulation (García-Cañedo et al., 2016), of which the conclusion is consistent with our data. Moreover, the significantly improved carotenoids also resulted in the increase of NPQ values in *N. oceanica* transformants. NPQ is a switchable mechanism to protect photosynthetic apparatus from photodamage caused by high light, and has been considered as an important parameter reflecting the accumulation of carotenoid whose function is to dissipate excess excitation energy as heat (Pirastru et al., 2012).

### Overexpressed *PtCRTISO4* enhances the violaxanthin contents of *Nannochloropsis oceanica*

Different with other photosynthetic eukaryotes, the predominant light harvesting complex of *Nannochloropsis* is violaxanthin–chlorophyll a binding protein (VCP), which binds abundant violaxanthin and minor vaucheriaxanthin as well as traces of zeaxanthin and antheraxanthin (Kean et al., 2016; Llansola-Portoles et al., 2017). The unique photosynthetic apparatus of *Nannochloropsis* underlies its peculiar pigment composition featured by extraordinarily high ratio of violaxanthin. According to the AlgaeBase, five species and several varieties belong to the genus of *Nannochloropsis*, and all of them have rich violaxanthin. For example, Safafar et al. (2015) evaluated the potential of microalgae as a source of natural antioxidants, and found that the violaxanthin of *N*. *salina* and *N*. *limnetica* were several times higher than other four species of Bacillariophyceae and Chlorophyta. In another study, the carotenoid profiles of 12 microalgae species were tested, and the results demonstrated that *Nannochloropsis* sp. BR2 ranked 2nd in terms of violaxanthin content (1.08 mg g^–1^ DCW) (Ahmed et al., 2014). *N*. *oculate* and *N*. *gaditana* have been stated to contain 1.2 and 3.37 mg g^–1^ DCW of violaxanthin contents, respectively (Neto et al., 2018; Lena et al., 2019). Wang and Jia systematically analyzed the impact of light intensity and growth stage on the pigment of *N*. *oceanica*, and observed that the violaxanthin peaked at Day 4 (5.6 mg g^–1^ DCW), and then declined along with the extending of cultivation time (Wang and Jia, 2020). Recently, Park et al. (2021) developed a mutant *N*. *oceanica* strain *via* γ-ray mutagenesis, the violaxanthin of which achieved 4.09 and 5.21 mg g^–1^ DCW under normal and optimal cultivation conditions, respectively.

Though the function of CRTISO has been addressed for decades (Isaacson et al., 2002, 2004), it has rarely been considered as the rate-limiting enzyme for carotenoid accumulation because its activity can be partially compensated by photoisomerization (Isaacson et al., 2002; Kenjiro et al., 2019). Consequently, compared to abundant research of *CRTISO* knock down/out, only one *CRTISO* overexpressed study has been performed to enhance the carotenoid accumulation very recently (Li et al., 2020). In that report, the authors introduced *PDS*, *ZDS*, and *CRTISO* of wolfberry into tobacco individually, and found that all three exogenous genes resulted in great increase of carotenoids, whereas the improvement magnitude caused by *CRTISO* was significantly higher than the other two genes, even though *PDS* had been recognized as the rate-limiting enzyme of carotenogenesis (Huang et al., 2008; Wang et al., 2009). In this study, the overexpression of *PtCRTISO4* remarkably enhanced the carotenoid contents of *N*. *oceanica*. Among all three overexpressed lines, T3 exhibited the highest total carotenoid content as well as violaxanthin content, and the latter reached up to 4.48 mg g^–1^ DCW. Compared with previous reports, the values obtained from T3 represented one of the richest violaxanthin contents known. Both our data and previous reports demonstrated a positive impact of *CRTISO* on carotenoid accumulation, and suggested that the overexpression of CRTISO is an effective strategy to develop outstanding *Nannochloropsis* strains featured by rich violaxanthin.

Vaucheriaxanthin is another key member of VCP, of which the molecules accommodated into the antenna complex are less than the violaxanthin but more than the zeaxanthin and antheraxanthin. However, vaucheriaxanthin is mainly present in the form of vaucheriaxanthin acyl esters (one of the chemotaxonomic markers of Eustigmatophyta) rather than free pigment (Stefania et al., 2014; Llansola-Portoles et al., 2017). In this study, the contents of vaucheriaxanthin acyl esters were failed to be determined due to the absence of standards. And thus, our vaucheriaxanthin values were much lower than the violaxanthin, and even below the zeaxanthin and antheraxanthin.

### *PtCRTISO4* plays a dual role in carotenegenesis

Both ZDS and CRTISO sequentially participate in the *cis*-carotenoid to *trans*-carotenoid conversions (from 9,9′-di-*cis*-ζ-carotene to all-*trans*-lycopene *via* 7,9,7′,9′-tetra-*cis*-lycopene and 7,9,7′,9′-tetra-*cis*-lycopene), and then LCYB gives rise to the dicyclic β-carotene and γ-carotene using all-*trans*-lycopene as substrate. Interestingly, it was *NoPSY*, catalyzing the distal upstream biosynthetic step of lycopene formation, that presented the highest transcriptional enhancements in the transformed lines, rather than *NoZDS* and *NoLCYB*, both of which were adjacent to *CRTISO* (Figure 1). Such an unusual observation implied that a feedback regulation pathway linked to *PSY* was possibly present in *N*. *oceanica* (Figure 1). In higher plants, it is known that the carotenoid biosynthesis can be modulated by various environmental factors (e.g., salt, light, and drought), and *PSY* genes are the most common regulatory sites (Nisar et al., 2015). For example, over 30 regulatory elements have been obtained from the *PSY1* promoter and its 5′-UTR sequence (Efremov et al., 2020), and a positive feedback loop between abscisic acid and root specific *PSY3* also has been observed from the Poaceae (Li et al., 2008). Recently, a novel metabolite-dependent feedback regulation loop linked to *PSY* has been proposed, in which pathway *cis*-carotenes (the substrates of CRTISO) and their derivatives (apocarotenoids) acted as the retrograde signals (Kachanovsky et al., 2012; Álvarez et al., 2016; Cazzonelli et al., 2020; Escobar-Tovar et al., 2020). Given that the accumulation of *cis*-carotenes is largely influenced by CRTISO (Cazzonelli et al., 2020), it can be speculated that the overexpression of *PtCRTISO4* induced the alteration of *cis*-carotenes concentrations as well as apocarotenoids, which further resulted in the significant enhancement of *PSY* expression by feedback regulation. In this case, the introduced *PtCRTISO4* exhibited a dual role in carotenogenesis: an isomerization role for the synthesis of all-*trans*-lycopene and a regulatory role linked to *PSY* expression. Such an inference explains the abnormal upregulation of *PSY* and large enhancement of carotenoid contents resulted from the overexpression of exogenous *CRTISO*.

The requirement of carotene isomerization for the assembly of PSII complexes has been observed from many organisms. For example, under light-activated heterotrophic growth conditions (5–10 min illumination daily), a Δ*crtH/B* double mutant of *Synechocystis* sp. (lacking the genes encoding CRTISO and phytoene synthase) lost the capability of producing carotenoids, what further led to strong suppression of CP43 and CP47 synthesis and the absence of oxygen evolving ability (Ozge et al., 2010). Similar phenomenon also has been observed from rice, the functional loss of CRTISO resulted in the transcriptional downregulation of CP43 and translational suppression of CP47 and D1 (Kazumori et al., 2004). The strengthened expression of CP43 and CP47 observed from the transformed lines of *Nannochloropsis* further suggests the presence of a coordinated regulation mechanism between CRTISO and reaction center proteins of PSII.

### Overexpressing *PtCRTISO4* has no negative effect on fatty acid accumulation but increases the C18:1

There are two different pathways: mevalonate (MVA) pathway and methylerythritol phosphate (MEP) pathway, responsible for the biosynthesis of IPP and DMAPP in microalgae (Gupta et al., 2021). In the MVA pathway, the first reaction is the condensation of acetyl-CoA, which is also the primary substrate of FA (Zhang et al., 2021). In other words, the MVA pathway for carotenoid synthesis may compete for acetyl-CoA with the *de novo* biosynthesis pathway of FA (Lee et al., 2019; Figure 1). Thus, the overexpression of carotenoid biosynthetic relevant genes is possible to rebalance the carbon flux between the FA biosynthesis and carotenoid accumulation. However, many microalgae, including *Nannochloropsis*, have lost the ancestral MVA pathway (Lu et al., 2014; Huang et al., 2021). Consequently, although the overexpression of *PtCRTISO4* sharply increased the carotenoid contents of *N*. *oceanica*, the FA contents of transformants still remained close to the WT.

It has been shown that the *de novo* biosynthesis of several xanthophylls is accompanied by esterification with FAs, during which process, specific FA will serve as a main feedstock (Holtin et al., 2009; Maoka, 2020; Gupta et al., 2021). Our results here suggest that C18:1 was likely the main type of FA participating in the esterification of carotenoids in *N*. *oceanica*. Interestingly, both C16:1 and C18:1 are synthesized by Δ9 desaturase using C16:0 and C18:0 as substrates, respectively. Up to now, there is no evidence to show the substrate specificity of Δ9 desaturase is adjustable, and thus we prefer to believe that the contrary trend exhibited by C16:1 and C18:1 is caused by the enzyme polymorphism. In fact, microalgae always possess several types of Δ9 desaturases showing different substrate preferences (Zhuang et al., 2022). For example, *Fistulifera* sp. has four Δ9 desaturases, two of which govern the formation of C16:1, while the other two can synthesis both C16:1 and C18:1 (Muto et al., 2013). In *Nannochloropsis*, several putative Δ9 desaturase have been supposed (Janssen et al., 2020), while only one of them showing a strong substrate preference toward C18:0 has been functionally verified (Zhang et al., 2021). All these studies and our results suggested the presence of other Δ9 desaturases possessing substrate specificity of C16:0 in *Nannochloropsis*.

### Advantages of *Nannochloropsis* as a eukaryotic expression system

Microalgae of Bacillariophyta contain chloroplasts surrounded by four membranes deriving from the secondary endosymbiosis event, meaning that their carotenoids biosynthetic enzymes which function in plastid require a relatively unique intracellular localization mechanism. In such a case, it seems difficult to directly perform the functional verification of these proteins using *Chlamydomonas* and *Chlorella* as eukaryotic expression systems. *Nannochloropsis* belongs to the phylum of Ochrophyta, of which the chloroplast is also enclosed by four membranes (Dolch et al., 2017). In this study, we did not make any modification toward the signal peptides of PtCRTISO4, and the widely altered phenotypes implied that the target gene had been localized correctly. Given the facts of the haploid genome, comprehensive genome engineering toolbox, and high genetic transformation efficiency (Ma R. et al., 2018; Park et al., 2021), *Nannochloropsis* can be taken as the optimal expression system to conduct the functional analysis for plastid-targeted genes of microalgae undergone secondary endosymbiosis.

## Conclusion

In summary, PtCRTISO4 was the most likely CRTISO of *P*. *tricornutum*. The introduction of *PtCRTISO4* remarkably improved the carotenoid accumulation of *N*. *oceanica*, generating one transformant with a high violaxanthin content of 4.48 mg g^–1^ DCW. The enhanced carotenoid further resulted in the increases of NPQ and ETR but not *F*_*v*_/*F*_*m*_. Interestingly, *NoPSY* was the most strengthened gene rather than *NoLCYB* and *NoZDS*, suggesting the presence of a feedback regulation loop between PSY and CRTISO. Majority of FA proportions remained similar between WT and transformants, nevertheless C16:1 and C18:1 were significantly reduced and improved, respectively. Such an increase trend of C18:1 implied that C18:1 might serve as a main feedstock for xanthophyll esterification in *N*. *oceanica*. Moreover, our results also implied that *Nannochloropsis* was an ideal expression system for the functional analysis of plastid-targeted genes of the microorganisms undergone secondary endosymbiosis.

## Data availability statement

The original contributions presented in this study are included in the article/Supplementary material, further inquiries can be directed to the corresponding author/s.

## Author contributions

YS, YX, and LYZ accomplished the gene isolation, transformation, positive transformants screening, and carotenoid analysis. CZ and XL supervised the experiments and provided guidance related to molecular manipulation. YW and RL contributed to the fatty acid analysis. LZ and JH designed the experiment, analyzed the data, and drafted the manuscript. All authors contributed to the article and approved the submitted version.
