# Supplementary material for: Enhancement of violaxanthin accumulation in Nannochloropsis oceanica by overexpressing a carotenoid isomerase gene from Phaeodactylum tricornutum
Source: Front Microbiol. 2022 Aug 31;13:942883. doi: 10.3389/fmicb.2022.942883 (PMC9471142; doi:10.3389/fmicb.2022.942883)
Supplement: Supplementary file 1 [file Data_Sheet_1.docx]

**Supplementary Table 1** Primers used for qRT-PCR

| Application/name | Protein ID | Primer sequence (5’–3’) | Size |
| --- | --- | --- | --- |
| Primers for qPCR of *P. tricornutum* | | | |
| F-*PtCdkA* | Phatr3_J20262 | CGAAGTCGTTACCCTGTGGT | 155 bp |
| R-*PtCdkA* |  | CCAATTGATCGGCTTCAGAT |  |
| F-*PtZEP2* | Phatr3_J5928 | GCATAGAATCAATACAAGGCACA | 162 bp |
| R-*PtZEP2* |  | GCAATAGCAATCTTCAGAGGC |  |
| F-*PtZEP3* | Phatr3_J10970 | GAGACAACCCACTACAACGG | 150 bp |
| R-*PtZEP3* |  | GCCAAGGAAAGACCACCAA |  |
| F-*PtVDL1* | Phatr3_J36048 | GACTCGGTTTCTCCATCGC | 128 bp |
| R-*PtVDL1* |  | TTGCTCCATTTGGTTATCTTG |  |
| F-*PtPSY* | Phatr3_EG02349 | AGTCGGGCACGATTTGGGTA | 134 bp |
| R-*PtPSY* |  | GAGCGTTGTCAGTTTGAGGTTGT |  |
| F-*PtCRTISO1* | Phatr3_J51868 | TATTGACGGATGGCACAGT | 115 bp |
| R-*PtCRTISO1* |  | CGTTTACTTCACCAGGCAC |  |
| F-*PtCRTISO2* | Phatr3_J54826 | ATACTTAACATGATTGCCGTCTC | 132 bp |
| R-*PtCRTISO2* |  | TCTTCGATCTAGTCCCTCCC |  |
| F-*PtCRTISO3* | Phatr3_J54842 | AACTCACCCAGCCATTTAG | 101 bp |
| R-*PtCRTISO3* |  | TGGTAACCCAGAAAGACAAAA |  |
| F-*PtCRTISO4* | Phatr3_J45243 | CTTAAACGCAAGAAAGTCAGG | 100 bp |
| R-*PtCRTISO4* |  | CAAATCGGCAATACCACCC |  |
| F-*PtCRTISO5* | Phatr3_J9210 | CCCCGCTCATTTAGTAGTTC | 123 bp |
| R-*PtCRTISO5* |  | TTGTAATCGTCGTACTCCTGT |  |
| F-*PtCRTISO6* | Phatr3_J42980 | TGCTGGGGAGTTATTGGTA | 150 bp |
| R-*PtCRTISO6* |  | GGGAATGAAAGACAGGACG |  |
| F-*PtCRTISO-L* | Phatr3_EG01981 | TCGTCGCCTAAAGGCTGTC | 132 bp |
| R-*PtCRTISO-L* |  | CGGACCAACGCAGTAGCAC |  |
| Primers for qPCR of *N. oceanica* | | | |
| F-*NoACT2* | NO03G02590.1 | ACCTTCTACAACGAGCTGC | 117 bp |
| R-*NoACT2* |  | GAACGTCTCAAACATAATCTGG |  |
| F-*NoTUA* | NO12G02410.1 | TGGCATGCTGCCTCATGTACC | 130 bp |
| R-*NoTUA* |  | TTGATGCCGCACTTGAAGC |  |
| F-*NoZEP2* | NO17G01820.1 | CGTGCGTATTGGAACTGGC | 187 bp |
| R-*NoZEP2* |  | TCCCTTATCCTCCCGAAAC |  |
| F-*NoPSY* | NO20G01840.1 | CAGCACGACGAGAAGAAGAAA | 156 bp |
| R-*NoPSY* |  | CGTGACGAAGGCAGTAGCA |  |
| F-*NoPDS* | NO16G01970.1 | CCCACGGTAGTCATTGCTG | 92 bp |
| R-*NoPDS* |  | CCTGGTAGGCGGACACTTT |  |
| F-*NoZDS* | NO03G05250.1 | GGTGCTCCTATCAATGGTCTC | 152 bp |
| R-*NoZDS* |  | TCTAACGCCCGAACATCCT |  |
| F-*NoLCYB* | NO10G02110.1 | GGGTGCCACGGTGCTCAAT | 100 bp |
| R-*NoLCYB* |  | ACCTCCAAAGGCCACGACT |  |
| F-*NoVDL* | NO11G00920.1 | GTTCCCTTGTCCTGTCCCA | 135 bp |
| R-*NoVDL* |  | TTCGTGCTTGTCCCTGTCG |  |
| F-*NoVDE* | NO24G00840.1 | TTTTCGAGAACGAGGTGGTG | 106 bp |
| R-*NoVDE* |  | TGCCCTTGAAGGAATTGATGT |  |
| F-*NoCP43* | KC598086^1^ (36197…37648) | AACGTCGAGCTGCTGAATA | 140 bp |
| R-*NoCP43* |  | CCATCCTAAGAAGAAATGAGAAC |  |
| F-*NoCP47* | KC598086^1^ (14472…16001) | CTGCTATTTGGCATTGGGTAT | 193 bp |
| R-*NoCP47* |  | AAACCGTAAGCATCTGATAGCC |  |

KC598086^1^: Accession number of NCBI, the numbers in the bracket indicate the locations of the corresponding gene.

**Supplementary Table 2** *C*_t_ values for candidate reference genes of *N. oceanica*

| Genes | WT | T1 | T2 | T3 | CV |
| --- | --- | --- | --- | --- | --- |
| *TUA* | 22.32 ± 0.13 | 23.37 ± 0.02 | 22.39 ± 0.04 | 24.41 ± 0.08 | 4.25% |
| *ACT1* | 22.64 ± 0.05 | 25.69 ± 0.05 | 24.48 ± 0.17 | 26.27 ± 0.08 | 6.48% |
| *ACT2* | 21.26 ± 0.06 | 24.30 ± 0.10 | 23.09 ± 0.12 | 24.09 ± 0.05 | 5.99% |

CV: Coefficient of variation.
